# Changes in Blood Lipid Levels After a Digitally Enabled Cardiometabolic Preventive Health Program: Pre-Post Study in an Adult Dutch General Population Cohort

**DOI:** 10.2196/34946

**Published:** 2022-03-23

**Authors:** José Castela Forte, Rahul Gannamani, Pytrik Folkertsma, Sridhar Kumaraswamy, Sarah Mount, Sipko van Dam, Jan Hoogsteen

**Affiliations:** 1 Department of Clinical Pharmacy and Pharmacology University Medical Centre Groningen Groningen Netherlands; 2 Ancora Health BV Groningen Netherlands; 3 Department of Neurology University Medical Centre Groningen Groningen Netherlands

**Keywords:** cholesterol, lifestyle intervention, prevention, hypercholesterolemia, digital health

## Abstract

**Background:**

Despite widespread education, many individuals fail to follow basic health behaviors such as consuming a healthy diet and exercising. Positive changes in lifestyle habits are associated with improvements in multiple cardiometabolic health risk factors, including lipid levels. Digital lifestyle interventions have been suggested as a viable complement or potential alternative to conventional health behavior change strategies. However, the benefit of digital preventive interventions for lipid levels in a preventive health context remains unclear.

**Objective:**

This observational study aimed to determine how the levels of lipids, namely total cholesterol, high-density lipoprotein (HDL) cholesterol, low-density lipoprotein (LDL) cholesterol, non-HDL cholesterol, and triglycerides, changed over time in a Dutch general population cohort undergoing a digital preventive health program. Moreover, we looked to establish associations between lifestyle factors at baseline and lipid levels.

**Methods:**

We included 348 adults from the Dutch general population who underwent a digitally enabled preventive health program at Ancora Health between January 2020 and October 2021. Upon enrollment, participants underwent a baseline assessment involving a comprehensive lifestyle questionnaire, a blood biochemistry panel, physical measurements, and cardiopulmonary fitness measurements. Thereafter, users underwent a lifestyle coaching program and could access the digital application to register and track health behaviors, weight, and anthropometric data at any time. Lipid levels were categorized as normal, elevated, high, and clinical dyslipidemia according to accepted international standards. If at least one lipid marker was high or HDL was low, participants received specific coaching and advice for cardiometabolic health. We retrospectively analyzed the mean and percentage changes in lipid markers in users who were remeasured after a cardiometabolic health–focused intervention, and studied the association between baseline user lifestyle characteristics and having normal lipid levels.

**Results:**

In our cohort, 199 (57.2%) participants had dyslipidemia at baseline, of which 104 participants were advised to follow a cardiometabolic health–focused intervention. Eating more amounts of favorable food groups and being more active were associated with normal lipid profiles. Among the participants who underwent remeasurement 9 months after intervention completion, 57% (17/30), 61% (19/31), 56% (15/27), 82% (9/11), and 100% (8/8) showed improvements at remeasurement for total, LDL, HDL, and non-HDL cholesterol, and triglycerides, respectively. Moreover, between 35.3% and 77.8% showed a return to normal levels. In those with high lipid levels at baseline, total cholesterol decreased by 0.5 mmol/L (7.5%), LDL cholesterol decreased by 0.39 mmol/L (10.0%), non-HDL cholesterol decreased by 0.44 mmol/L (8.3%), triglycerides decreased by 0.97 mmol/L (32.0%), and HDL increased by 0.17 mmol/L (15.6%), after the intervention.

**Conclusions:**

A cardiometabolic screening program in a general population cohort identified a significant portion of individuals with subclinical and clinical lipid levels. Individuals who, after screening, actively engaged in a cardiometabolic health–focused lifestyle program improved their lipid levels.

## Introduction

The morbidity and mortality burden associated with cardiovascular disease (CVD) continues to increase globally [1]. With prevalent cases of CVD having nearly doubled since 1990 to almost 523 million cases worldwide, it is now the leading cause of global mortality and a major contributor to disability [1]. The etiology of CVD is multifold, including genetic predisposition, socioeconomic and environmental factors, and lifestyle [2]. In fact, approximately 50% of CVD risk is attributable to modifiable lifestyle factors, such as an unhealthy diet, lack of physical activity, and smoking, which subsequently lead to metabolic imbalances and overweight or obesity [1-3].

Dyslipidemia, defined as elevated levels of total cholesterol, low-density lipoprotein (LDL) cholesterol, or triglycerides, or low levels of high-density lipoprotein (HDL) cholesterol, is a major risk factor for CVD [4]. As with other CVD risk factors, genetic risk plays a role in the development of dyslipidemia, such as in familial hypercholesterolemia; however, the majority of cases are due to unhealthy lifestyle behaviors [5,6]. As such, lifestyle interventions are central to dyslipidemia prevention and are recommended for all patients before pharmacotherapy is prescribed, and even after pharmacotherapy initiation [5]. Lifestyle changes that have been shown to be beneficial for dyslipidemia are simple and well-known to the general public, such as following a diet emphasizing the intake of vegetables, fruits, legumes, and whole grains, and minimizing the intake of processed meats, refined carbohydrates, and sweetened beverages, as well as doing sufficient daily low-intensity activity [7-9]. Although most national and international guidelines consider both healthy lifestyle behaviors and preventive medication as cornerstones of CVD primary and secondary prevention, there is a lack of effective strategies promoting risk reduction through these lifestyle factors [9,10]. This is because primary care providers often struggle to implement advice and referral structures for lifestyle promotion and individuals fail to successfully change and maintain favorable health behaviors that modify these risk factors [11-13]. The reasons for the latter vary greatly from limiting social constructs, such as work hours, family duties, and socioeconomic factors, to personal factors, such as low self-efficacy, motivation, and lack of perceived benefit [14,15].

A growing number of digital application–based programs that can support individuals in addressing these challenges are being developed and are both publicly and commercially available [16,17]. Previous studies have demonstrated the benefits of digital applications for improving medication adherence and reducing cardiovascular risk in patients at higher cardiovascular risk and in patients living with CVD [18-20]. Therefore, these applications can broaden access to prevention strategies and care outside of traditional care [20]. Yet, there is scarce robust data on the effectiveness of such applications for modulating lifestyle-related risk factors, such as lipid profiles, and few, if any, studies have demonstrated the effects of a digitally enabled lifestyle intervention in a presumably healthy general population cohort [21,22].

The Ancora Health platform is a digital application that supports a preventive health screening and lifestyle coaching intervention. Individuals undergo a health assessment, and receive a Personal Health Passport (PHP) with their data and the outline of the intervention. Then, they go through a 16-week coaching program, initiated with a 30-minute video consultation by a medical doctor. This initiation session consists of counselling on health insights (risks in aspects of physical and mental health); recommendation of targeted lifestyle medicine actions (which can also be tracked by individuals in their PHP); and getting a buy-in to undertake these actions for the following period. Coaching is primarily digital, one-on-one, chat-based digital coaching (optional audio/video call alongside this) from either a lifestyle coach, personal trainer, dietitian, or psychologist. This is complemented by weekly progress reports with feedback. Through this approach, participants are provided peer-support and motivation, are coached on how to acquire and maintain healthy habits, learn how to overcome barriers encountered during behavior change, and receive tips/tricks on how to implement new behaviors into daily practice.

In this study, we assessed the prevalence of dyslipidemia in a Dutch general population cohort undergoing health screening, and measured the effect of the subsequent digitally enabled lifestyle program on participants’ lipid profiles in the first cohort of individuals undergoing this program. 

## Methods

### Study Sample

**Figure 1:**
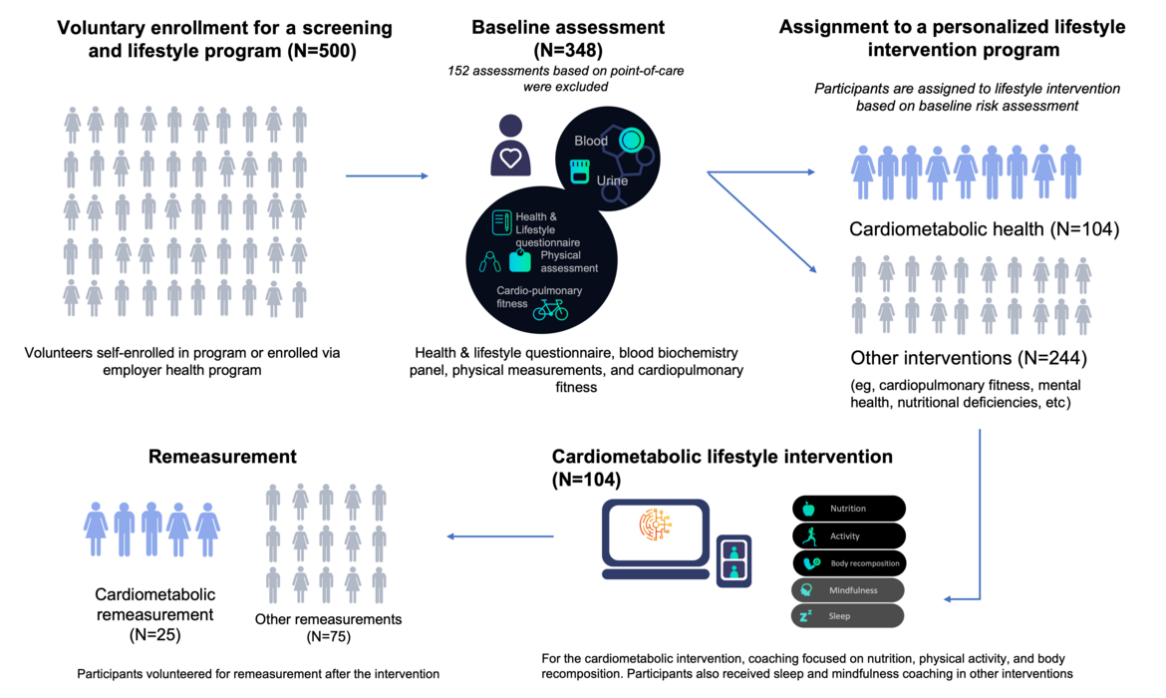
Overview of the study flow, including sample size at each stage. Changes in lipid values presented in the results are derived from the cardiometabolic remeasurement group (N=25).

As of October 2021, more than 500 users had enrolled in an Ancora Health Lifestyle program, with many getting their lipid markers (total cholesterol, HDL cholesterol, LDL cholesterol, non-HDL cholesterol, total/HDL cholesterol ratio, and triglycerides) measured through venipuncture. Of those, 100 users also came for a remeasurement after concluding the program. We excluded participants whose first or second measurement was done using a point-of-care device. Our final sample size was 348 participants who had their blood lipid levels determined via venipuncture at both timepoints. An overview of the study flow is given in Figure 1.

### The Platform

The Ancora Health PHP is a certified Class I medical device that presents individuals their current health status and possible future health risks based on a broad assessment of body, mind, and lifestyle. Participants undergo a physical intake, where blood biomarkers, including cardiometabolic markers, physical measurements, and cardiopulmonary fitness measurements are assessed. Additionally, individuals provide diet- and activity-related information through a Health and Lifestyle Questionnaire, as well as previous medical and family history. The PHP uses these inputs to stratify individual risk, placing individuals on a gradient between health (no elevated risk based on the absence of measurable risk factors) and disease (beyond clinical threshold based on clinical guidelines). Based on this stratification, participants are provided lifestyle coaching on nutrition, physical activity, and other health behaviors. The goal of the program is to create lasting behavior change through motivation, education, and personalized recommendations, to modulate the identified modifiable risk factors.

The Ancora Health application is available in The Netherlands directly to the consumer, and through selected health plans and employers.

### Measurements at Intake, During the Program, and After the Program

Upon enrollment to the program, participants underwent a baseline assessment involving a comprehensive lifestyle questionnaire, a blood biochemistry panel, physical measurements, and cardiopulmonary fitness measurements. After the baseline assessment, users could access the digital web application to register and track their health behaviors, and modify weight and anthropometrics data at any time during the intervention. At follow-up after the intervention, the subset of blood biochemistry parameters found to be abnormal at baseline, and the lifestyle questionnaire and physical measurements, with or without cardiopulmonary fitness assessment, were remeasured.

We defined the following prespecified cutoffs for all lipid markers: normal, elevated, high, and clinical threshold for dyslipidemia, which, if crossed, would result in advice to discuss the findings with a care practitioner. For total cholesterol, the thresholds for “normal,” “elevated,” “high,” and “clinical dyslipidemia” were <5.1 mmol/L, 5.1-6.2 mmol/L, 6.2-8.0 mmol/L, and ≥8 mmol/L (the clinical threshold leading to referral), respectively; for LDL cholesterol, the same thresholds were <3.0 mmol/L, 3.0-4.1 mmol/L, 4.1-4.9 mmol/L, and ≥4.9 mmol/L, respectively; and for triglycerides, the same thresholds were <1.8 mmol/L, 1.8-2.3 mmol/L, 2.3-5.6 mmol/L, and ≥5.6 mmol/L, respectively [23,24]. For HDL cholesterol, levels <1 mmol/L were considered low, those between 1 and 1.2 mmol/L were considered suboptimal, those between 1.2 and 2.3 mmol/L were considered normal, and those above 2.3 mmol/L were considered elevated [25]. The same thresholds were applied to the remeasured values at follow-up. Not all individuals with dyslipidemia followed a cardiometabolic health–focused intervention. Only individuals in whom at least one marker was high or HDL cholesterol was low received specific coaching and advice. For instance, individuals with low HDL cholesterol received advice to consume more healthy fats (ie, fatty fish, nuts and seeds, and avocado, depending on their dietary restrictions), while individuals who needed to reduce total or LDL cholesterol were advised to consume more fiber-rich foods and limit saturated and transsaturated fats, and were coached specifically on how to implement and maintain these nutritional habits. Others with normal or only elevated lipid levels underwent an intervention with coaching on a variety of other aspects, from mental health to endurance training. In these cases, no specific cardiometabolic advice was given unless proactively requested by the participant.

Changes in lipid markers from baseline were calculated by subtracting the first reported values from the end values, and the percentage change was calculated by dividing the observed change by the baseline value. We also examined values of the cholesterol ratio (total/HDL cholesterol), with a threshold of ≥5 considered elevated [26]. BMI was calculated at baseline and remeasurement as weight in kilograms divided by height in meters squared (kg/m^2^).

### Dietary Assessment

Food group consumption was assessed by means of web-based weekly dietary questionnaires, filled in upon enrollment in the program and at remeasurement. Thresholds for unusually low or high portion sizes were defined a priori per food group based on the Dutch Nutritional Guidelines, with the number of portions per week being entered as multiple choice, to minimize incorrect entries. Changes in food group consumption were calculated as the difference between baseline and remeasured self-reported consumption. Participants were assigned a classification between insufficient and excessive consumption for food groups seen as favorable or neutral for improving lipid profiles (pulses and beans, fatty fish, dark chocolate, coffee or tea, low fat dairy, whole grain foods, fruit, leafy greens, herbs, nuts and seeds, poultry, unsaturated fats and oils, meat substitutes, shellfish, soy products, and lean fish) and food groups unfavorable to lipid profiles (eggs, full-fat dairy, red meat, processed meat, sweetened beverages, refined grains, saturated fats and oils, sweets, and fast food) based on national guidelines and the literature [27,28].

### Statistical Analysis

Descriptive statistics were calculated to characterize the population at baseline, in terms of demographics and lipid markers. Additional analyses were conducted in the group of patients who had elevated or high lipid levels at baseline and subsequently underwent remeasurement. In this group, we calculated the mean start value, mean end value, and mean absolute and percentage changes of total cholesterol, total/HDL cholesterol ratio, HDL cholesterol, LDL cholesterol, and triglycerides. All categorical variables were reported as percentage (%) and continuous variables were reported as mean and SD. For differences in categorical variables, the chi-square test was used, and the analysis of variance test was used for continuous variables. We considered a *P* value <.05 as statistically significant for differences in biomarkers. All data analyses were performed using R software v4.0.3 (The R Project for Statistical Computing). We also computed the percentage of participants by category of change in lipid parameters from baseline to after the intervention period, from clinical threshold values to normal values. The Pearson linear correlation factor, R, was used to assess the linear associations of baseline food group consumption, physical activity (self-reported low/moderate and high-intensity physical activity, as well as strength training), and type of occupational activity (sedentary or active in different extents) with cholesterol levels. *P* values for the associations of dietary factors and other lifestyle factors with lipid levels were adjusted for multiple comparisons.

### Ethics Statement

The study was declared exempt from institutional review board approval through a waiver issued by the Medical Ethical Committee of the University Medical Centre Groningen (waiver number: METC#2021/488). All analyses were performed in accordance with relevant guidelines and regulations.

## Results

### Baseline Characteristics

Baseline characteristics of the total study sample are shown in Table 1. We found that 199 participants (57.2%) had dyslipidemia at baseline, of which 39 (19.6%) crossed the clinical threshold. Additionally, 104 of the 199 had at least one high lipid marker or low HDL, and were therefore advised to follow a cardiometabolic health–focused intervention. Nine to 10 months after completion of the intervention, 100 participants underwent a remeasurement, of which 25 had partaken in the cardiometabolic health–focused intervention. Participants from this cardiometabolic subgroup were older, had higher lipid levels, and had higher weight and BMI compared to participants following interventions with other focuses, such as mental health and endurance training (Table 1).

**Table 1:** Baseline characteristics of the total study sample.

Characteristic		Baseline (N=348)	Cardiometabolic intervention group^a^ (N=104)	*P* value^b^
**Demographics**				
	Age (years), mean (SD)	44.6 (11.1)	49.4 (9.0)	.02
	Sex (female), n (%)	195 (56.0%)	38 (36.5%)	.09
**Anthropometrics**				
	Weight (kg), mean (SD)	77.2 (14.4)	83.5 (12.2)	.02
	BMI (kg/m^2^), mean (SD)	25.0 (4.7)	26.6 (2.9)	.01
	Body fat percentage, mean (SD)	24.9 (9.8)	26.7 (7.7)	.26
**Lipids**				
	Total cholesterol level (mmol/L), mean (SD)	5.10 (1.06)	6.00 (1.01)	<.001
	LDL^c^ cholesterol level (mmol/L), mean (SD)	3.13 (0.94)	4.19 (0.84)	<.001
	Triglyceride level (mmol/L), mean (SD)	1.12 (0.69)	1.88 (1.30)	.007
	HDL^d^ cholesterol level (mmol/L), mean (SD)	1.60 (0.42)	1.33 (0.40)	.003
	Total/HDL cholesterol ratio, mean (SD)	3.37 (1.12)	4.65 (1.09)	<.001
	Non-HDL cholesterol level (mmol/L), mean (SD)	3.49 (1.07)	4.64 (0.89)	<.001

^a^Participants with high lipid values at baseline who underwent a cardiometabolic health–focused intervention.

^b^Unpaired *t* test between the entire cohort and the cardiometabolic remeasurement group.

^c^LDL: low-density lipoprotein.

^d^HDL: high-density lipoprotein.

### Baseline Lipid Levels and Association With Lifestyle Factors

Of 348 individuals at baseline, 199 (57.2%) had dyslipidemia. In particular, 162 users (46.6%) had elevated or high total cholesterol, 172 (49.4%) had elevated or high LDL cholesterol, 36 (10.3%) had elevated or high triglycerides, and 54 (15.5%) had low or suboptimal HDL cholesterol. More than half of these individuals (104/199, 52.3%) had at least one relevantly abnormal lipid marker, with 53 (15.2%) having high total or LDL cholesterol and 54 (15.5%) having low to suboptimal HDL cholesterol; high triglycerides were found in 22 (6.3%) participants. In addition to these, 39 (11.2%) participants were found to have at least one lipid marker beyond the clinical threshold: 2 (0.6%) with total cholesterol above 8 mmol/L, 18 (5.2%) with LDL cholesterol above 4.9 mmol/L, 18 (8.9%) with a total/HDL cholesterol ratio ≥5, and 1 (0.3%) with triglycerides above 5.6 mmol/L.

**Figure 2:**
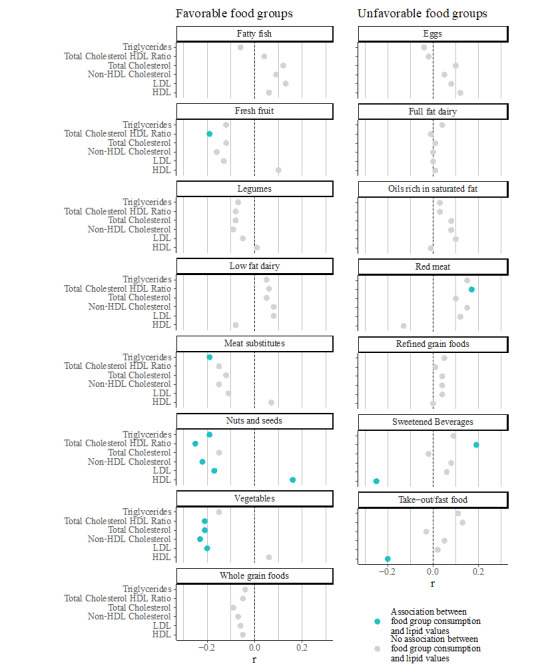
Associations between baseline food group consumption and lipid levels at baseline. HDL: high-density lipoprotein; LDL: low-density lipoprotein.

The regression analysis between food group consumption and baseline lipid levels revealed a significant positive correlation between the consumption of several favorable food groups and having normal lipid levels (Figure 2). Consuming more portions of nuts and seeds was associated with a better lipid profile across all markers, with significant associations for all markers other than total cholesterol (R=−0.19 to −0.21; *P*<.001). Consuming more fresh fruits was associated with better lipid values across the board, and was significantly associated with an improved total/HDL cholesterol ratio (R=−0.15; *P*=.03). Higher vegetable consumption was also highly associated with normal lipid levels across all markers except triglycerides (R=−0.20 to −0.23; *P*<.001). On the other hand, consumption of unfavorable food groups was associated with higher lipid levels. Higher red meat consumption was associated with higher lipids across the board, especially the total/HDL cholesterol ratio (R=0.17; *P*<.001). Consuming more take-out/fast food was also associated with higher triglycerides (R=0.11; *P*=.03) and lower HDL cholesterol (R=−0.19; *P*<.001). Interestingly, consumption of sweetened beverages was markedly associated with lower HDL cholesterol and a higher total/HDL cholesterol ratio (R=−0.25 and 0.19, respectively; *P*<.001).

**Figure 3:**
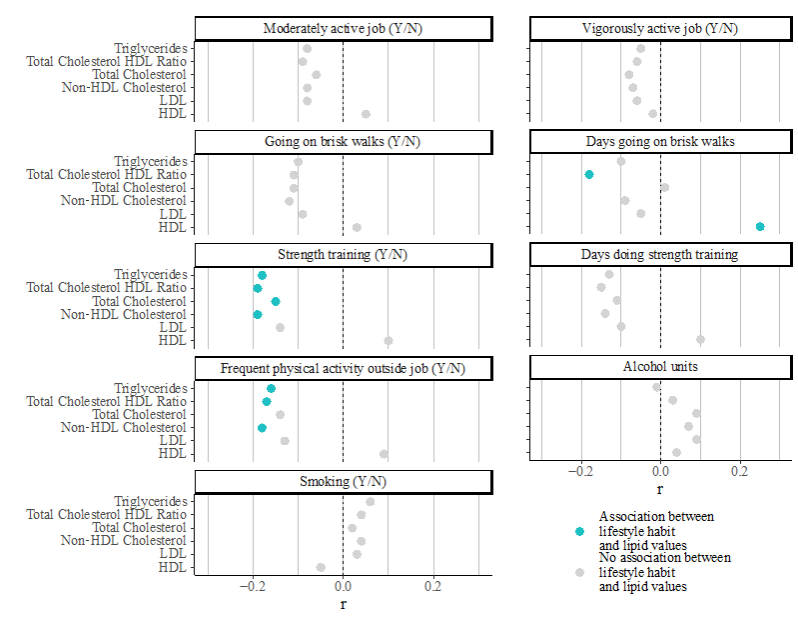
Associations of baseline physical activity levels and other lifestyle factors with lipid levels at baseline. HDL: high-density lipoprotein; LDL: low-density lipoprotein.

For physical activity, an association was found between more days of brisk walking and HDL cholesterol (R=0.25; *P<*.001) and the total/HDL cholesterol ratio (R=−0.18; *P*<.001) (Figure 3). Additionally, there was an association between doing or not doing strength training and lower total cholesterol, non-HDL cholesterol, and triglyceride levels (R=−0.19 to −0.15; *P*=.005). The number of days doing strength training per week showed no further association. Lastly, doing frequent physical activity outside working hours was also associated with normal lipid values (R=−0.17 to −0.12; *P*=.003).

### Changes in the Lipid Profile

There were 30 participants with elevated or high total cholesterol levels at baseline who underwent remeasurement. Of these, 17 (57%) showed a decrease at remeasurement, with 65% (11/17) showing at least a meaningful decrease and 35% (6/17) returning to within the normal range. On average, the total cholesterol reduction for those who underwent the intervention focused on cardiometabolic health was 0.50 (SD 0.71) mmol/L (*P*=.01; Table 2). There were 31 participants with elevated or high LDL cholesterol levels at baseline who underwent remeasurement. Of these, 19 (61%) showed a decrease at remeasurement, with 68% (13/19) showing at least a meaningful decrease and 37% (7/19) returning to within the normal range. In the cardiometabolic intervention group, this translated to a mean decrease of 0.30 (SD 0.59) mmol/L in LDL cholesterol after follow-up (*P*=.04; Table 2). Accordingly, significant differences were also found for non-HDL cholesterol, where of the 27 participants with elevated or high non-HDL cholesterol levels at baseline who underwent remeasurement, 15 (56%) showed a decrease at remeasurement, with 67% (10/15) showing at least a meaningful decrease and 47% (7/15) returning to within the normal range. On average, the non-HDL cholesterol reduction was 0.44 (SD 0.74) mmol/L (*P*<.05; Table 2). Eleven remeasured participants had abnormal HDL cholesterol levels at baseline. Of these, 9 (82%) showed an improvement at remeasurement, with 89% (8/9) showing at least a meaningful improvement and 78% (7/9) returning to within the normal range (*P*<.001; Table 2). Lastly, 8 participants with elevated or high triglyceride levels at baseline underwent remeasurement. All 8 participants showed a decrease in triglycerides, with 88% (7/8) showing at least a meaningful decrease and 50% (4/8) returning to within the normal range. In the intervention group, the average reduction was 0.97 (SD 0.31) mmol/L (*P*=.02).

**Table 2:** Changes in the lipid profile after the cardiometabolic intervention.

Variable	Value before the intervention	Value after the intervention	Absolute and relative (%) change	*P* value^a^
Total cholesterol level (mmol/L)	6.68	6.18	−0.50 (7.5%)	.01
LDL^b^ cholesterol level (mmol/L)	4.39	4.00	−0.30 (6.9%)	.04
Triglyceride level (mmol/L)	3.02	2.05	−0.97 (32.1%)	.02
HDL^c^ cholesterol level (mmol/L)	1.09	1.26	0.17 (15.6%)	<.001
Total/HDL cholesterol ratio	3.7	3.6	−0.1 (2.7%)	.31
Non-HDL cholesterol level (mmol/L)	5.31	4.87	−0.44 (8.3%)	.045

^a^Paired *t* test.

^b^LDL: low-density lipoprotein.

^c^HDL: high-density lipoprotein.

## Discussion

### Principal Findings

In this study, of 348 users participating in a digitally enabled combined lifestyle intervention program, we found that 57.2% had dyslipidemia, 29.9% had at least one relevantly abnormal lipid marker, and 8.9% had a lipid marker crossing a clinical threshold requiring referral. Eating more amounts of favorable food groups and being more active were associated with normal lipid profiles. Of those who had their levels remeasured after the intervention, more than 56% showed a decrease at remeasurement, and between 35.3% and 77.8% showed a return of the levels to within the normal range. These preliminary findings suggest that participating in a digitally enabled lifestyle intervention targeting behavioral change across multiple lifestyle factors associated with abnormal lipid levels leads to improvements in lipid markers. This may therefore be a scalable approach to cardiometabolic risk reduction at the population level.

### Comparison With Prior Work

The positive effect of lifestyle programs on cholesterol levels is well established, with reductions in total and LDL cholesterol varying from 7% to 20% and increases in HDL cholesterol varying from 10% to 15%, using interventions of different intensities and complexities [29,30]. Evidence for digital interventions targeting the reduction of cardiometabolic risk and CVD has been accumulating over the last 5 to 10 years, yet it is still sparse. A recent review showed that primarily mobile digital interventions targeting populations with high cardiovascular risk led to meaningful decreases in total and LDL cholesterol [20]. In line with this, a 2015 review and meta-analysis had already shown the great potential of these interventions to positively impact risk factors for CVD and subsequently also reduce CVD outcomes [31]. Across a variety of studies, reductions in CVD outcomes of up to 40% were documented, mediated by decreases in individual risk factors such as weight, BMI, and lipids. In particular, total cholesterol and LDL cholesterol improved on average by 0.13 to 0.14 mmol/L, while triglycerides showed no improvement. This provides a good comparison metric to the intervention deployed in our study, since in this review, most interventions were web-based. The reductions in LDL and total cholesterol achieved in our study were thus more substantial than those reported in the review, likely due to the blended nature of the intervention (eHealth combined with human coaching), rather than the primarily educational character of previous interventions. In fact, the effect of this web-based intervention was more comparable to studies focused on nutritional and/or exercise coaching provided via a mobile platform [20].

Since the first step of a targeted preventive health program is to stratify risk and define which modifiable parameters individuals should focus on, in this study, we also analyzed which lifestyle factors assessed at baseline were associated with abnormal lipid levels. We found that higher consumption of food groups generally seen as favorable for cardiovascular risk and, in particular, lipid level reduction was indeed associated with lower baseline lipid levels and a higher HDL cholesterol level. Evidence for the role of plant-based or low-meat diets in reducing cholesterol levels is increasing, with diets, such as the Portfolio Diet from the early 2000s, being shown to lower LDL cholesterol and CVD risk [32]. This is potentially mediated through an increase in dietary fiber consumption associated with plant-rich or low-meat diets, and is in line with our findings that higher consumption of legumes and vegetables was linked to lower levels of most lipid parameters. Moreover, some, but not all, food groups with anti-inflammatory properties, such as nuts and seeds or fatty fish rich in monounsaturated and polyunsaturated fats, were associated with higher HDL cholesterol levels [33,34]. The opposite was also true for food groups usually seen as unfavorable, with consumption of, for example, oils rich in saturated fat and red meat being associated with higher baseline levels of most lipid parameters, which is in line with the findings of previous studies that established a link between consumption of these food groups and CVD risk and mortality [35,36]. For triglycerides, associations were less present, though higher consumption of food groups, such as legumes and meat substitutes, was associated with lower levels. Interestingly, we did not find higher consumption of certain food groups, such as eggs, to be associated with CVD, despite previous epidemiological evidence of this [37]. Overall, despite the association between nutritional habits, such as consumption of certain food groups, and lipid levels having long been established, we showed that even in an otherwise healthy general population cohort, nutritional habits are associated with high preclinical lipid levels and therefore constitute a prime target for digitally enabled combined lifestyle interventions geared at reducing cardiometabolic risk factors and adding healthy life years to participants’ lives.

The identification of critical nutritional habits associated with higher lipid levels and cardiovascular risk is, however, only the first step. We showed that providing tailored nutritional and physical activity advice and coaching through a digital lifestyle platform resulted in measurable improvements in lipid levels. Few previous studies have demonstrated this, with a handful of studies summarized in reviews showing that interventions applying theoretical frameworks or models for behavioral change, some of which were technology based, were more effective at increasing adherence to healthy lifestyle habits than standard advice [38,39]. In particular, this framework led to improved cholesterol levels when used for cardiometabolic risk management [40]. Two studies reported on the effectiveness of nutrition-only platforms. In one, the effect of a digital nutritional tracking and meal planning platform on lipid markers in individuals with dyslipidemia was assessed, showing improvement in all parameters [41]. In the second study, a mixture of online and offline engagement was used, with participants who underwent the complete prevention program showing improved cholesterol levels [42]. Additionally, one study analyzed the effect of a mobile app providing health education and step counting on multiple cardiovascular risk factors in a presumably healthy population [43]. In the study, an increase in the daily step count attributed to the use of the app was associated with a reduction of 0.07 mmol/L in LDL cholesterol and an increase of 0.05 mmol/L in HDL cholesterol [43]. With this study, we contribute to the, for now, scarce body of evidence on the usefulness of digital lifestyle programs for cardiovascular risk reduction through improvement in lipid profiles. Together with the other studies in clinical populations, the findings from this study, which was focused on individuals with elevated lipid levels without overt dyslipidemia, underscore the potential usefulness of lifestyle interventions, especially when delivered digitally, for both the primary and secondary prevention of cardiometabolic risk. In fact, effective digital lifestyle interventions appear to magnify the therapeutic benefit of cholesterol-lowering medication in those already past the clinical threshold and receiving medication, because of the complementary effects of the pharmacological and lifestyle interventions [44].

Lastly, we need to address the point of the high attrition rate registered for the intervention. Of the 104 individuals stratified to the cardiometabolic health intervention, only 25 opted to be remeasured within the reported study period. Attrition rates in mobile health (mHealth) studies and real-world applications are known to be high, with previous research having identified that up to 80% of all participants in mHealth interventions may engage in only minimal use of these interventions and that the lowest user dropout rate was 40%, even in a trial setting [45,46]. The results presented here are, as stated, preliminary, and there is certainly room for improvement in the digital engagement strategies deployed in this version of the intervention. For example, the intervention will shortly be released in mobile app form, which will support more high quality and more frequent digital engagement due to integrations with suites, such as Apple Health Kit, Google Fit, and more [47]. However, despite these limitations, we consider the results reported for the subpopulation that underwent remeasurement to be sufficient to provide an answer to the research question we set out to investigate, namely, whether a digital lifestyle intervention can improve cardiometabolic risk factors in engaged individuals.

### Limitations

This study has several limitations. First, the sample size of the remeasured population was small. Second, there may be some selection bias in this remeasured population, as remeasurement was optional and voluntary. Participants who came for a remeasurement could represent a more engaged subpopulation or represent a group who actively worked on behavioral change and therefore expected results. Third, participants were required to return to the health center for a remeasurement of blood chemistry outcome parameters. This prevented us from assessing the changes verified in those who did not remeasure but may have nonetheless conducted lifestyle changes. We are currently evaluating several possibilities to overcome this. On the one hand, we are considering adapting our digital infrastructure to allow for input of self-measured blood values. However, this possibility needs to be weighed against the risk of inaccurate measurements due to interlaboratory analytical variability. On the other hand, we are studying options to structurally offer remeasurements at fixed time points. We believe this will help overcome the low remeasurement rate, which is likely to stem from the culture of health checks every 2 years in the Netherlands, both self-initiated and offered through employer health plans. Lastly, we did not account for socioeconomic factors, which are potential confounders in assessing the effect of the intervention.

Conversely, one strength of the study is that medication information was gathered at baseline and follow-up. This allowed us to verify that participants with improved lipid levels did not initiate cholesterol-lowering medications. In addition, few studies have reported on real-world applications of digital interventions targeting health behavior change and the effects on health parameters such as lipid levels. Using a database of users of the Ancora Health platform, we evaluated real-world data to analyze the changes in lipid levels before and after a digitally enabled lifestyle intervention, and find associations that support the usefulness and effectiveness of commercial digital applications of health behavioral change for cardiometabolic risk factor reduction. Additionally, the data gathered in the database spanned across multiple lifestyle domains. Few other studies reported on a broad range of lifestyle factors and their influence on lipid levels.

### Conclusions

While the positive effects of healthy lifestyle changes on lipid levels are well established in the literature, this is one of the first studies to examine changes in lipid profiles among individuals of the general population participating in a digitally enabled lifestyle program with personalized dietary and physical activity recommendations delivered through the combination of eHealth and human coaching protocols rooted in behavioral science. We confirmed findings from previous studies regarding the link between certain nutritional patterns/other health behaviors and lipid levels, even in a general population cohort. Importantly, we showed that digital interventions can achieve lipid level reductions comparable to other traditional lifestyle interventions. A high rate of attrition remains a potential problem for mHealth interventions, primarily those that are web-based, which may limit adoption at scale. These preliminary findings contribute to expanding the body of evidence on the potential of digital therapeutic platforms providing lifestyle coaching for improving lipid levels and thereby contribute to cardiovascular risk reduction.

## Abbreviations

CVD: cardiovascular disease
HDL: high-density lipoprotein
LDL: low-density lipoprotein
mHealth: mobile health
PHP: Personal Health Passport
